# A Novel Risk Factor Model Based on Glycolysis-Associated Genes for Predicting the Prognosis of Patients With Prostate Cancer

**DOI:** 10.3389/fonc.2021.605810

**Published:** 2021-09-14

**Authors:** Kaixuan Guo, Cong Lai, Juanyi Shi, Zhuang Tang, Cheng Liu, Kuiqing Li, Kewei Xu

**Affiliations:** ^1^Department of Urology, Sun Yat-sen Memorial Hospital, Sun Yat-sen University, Guangzhou, China; ^2^Guangdong Provincial Key Laboratory of Malignant Tumor Epigenetics and Gene Regulation, Sun Yat-sen Memorial Hospital, Sun Yat-sen University, Guangzhou, China

**Keywords:** glycolysis, prostate cancer, biochemical recurrence, prognosis, risk score, prediction model

## Abstract

**Background:**

Prostate cancer (PCa) is one of the most prevalent cancers among males, and its mortality rate is increasing due to biochemical recurrence (BCR). Glycolysis has been proven to play an important regulatory role in tumorigenesis. Although several key regulators or predictors involved in PCa progression have been found, the relationship between glycolysis and PCa is unclear; we aimed to develop a novel glycolysis-associated multifactor prediction model for better predicting the prognosis of PCa.

**Methods:**

Differential mRNA expression profiles derived from the Cancer Genome Atlas (TCGA) PCa cohort were generated through the “edgeR” package. Glycolysis-related genes were obtained from the GSEA database. Univariate Cox and LASSO regression analyses were used to identify genes significantly associated with disease-free survival. ROC curves were applied to evaluate the predictive value of the model. An external dataset derived from Gene Expression Omnibus (GEO) was used to verify the predictive ability. Glucose consumption and lactic production assays were used to assess changes in metabolic capacity, and Transwell assays were used to assess the invasion and migration of PC3 cells.

**Results:**

Five glycolysis-related genes were applied to construct a risk score prediction model. Patients with PCa derived from TCGA and GEO (GSE70770) were divided into high-risk and low-risk groups according to the median. In the TCGA cohort, the high-risk group had a poorer prognosis than the low-risk group, and the results were further verified in the GSE70770 cohort. *In vitro* experiments demonstrated that knocking down HMMR, KIF20A, PGM2L1, and ANKZF1 separately led to less glucose consumption, less lactic production, and inhibition of cell migration and invasion, and the results were the opposite with GPR87 knockdown.

**Conclusion:**

The risk score based on five glycolysis-related genes may serve as an accurate prognostic marker for PCa patients with BCR.

## Introduction

Prostate cancer (PCa) is the second most common cause of male malignant tumors and the second leading cause of tumor-associated death in males worldwide (1, 2). According to statistics, there were 1.3 million new cases and 359,000 related deaths worldwide in 2018 (1, 3, 4). PCa with biochemical recurrence (BCR) was a critical lethal factor that frequently led to a poor prognosis and seriously threatened patient survival (5). Although the concept of personalized management of BCR patients has been greatly improved, a superficial understanding of the molecular mechanisms of the disease severely limits clinicians’ treatment strategies, resulting in increased mortality of patients with BCR (6). Hence, there is an urgent need to develop an instructive tool to accurately judge the prognosis of PCa.

Aberrant switching between oxidative phosphorylation (OXPHOS) and glycolysis plays a crucial regulatory role in tumorigenesis (7–10). Initially, glycolysis was considered as a manner of energy supply forced adopted in the hypoxic microenvironment caused by uncontrolled growth (11). Interestingly, cancer cells prefer to utilize aerobic glycolysis to obtain adenosine triphosphate (ATP) to satisfy the needs for uncontrolled proliferation even in conditions with sufficient oxygen. This unique metabolic signature termed Warburg effect has been demonstrated to exist in various solid tumors, including PCa (12–14). Recently, an increasing number of studies have reported that abnormal expression of glycolysis-related molecules have crucial roles in regulating chemotherapy resistance (15), immune response (16, 17), neuroendocrine differentiation (18), and growth and metastasis of PCa (19, 20), ultimately affecting its prognosis. Additionally, some researchers have successfully constructed prediction models derived from glycolysis-associated genes in liver cancer and colon cancer (4, 21). However, the actual relationship between glycolysis and PCa remains unclear and is worth further exploration. Herein, we hypothesized that a risk score prediction model based on glycolysis-related genes might have a strong ability to accurately judge the prognosis of PCa with BCR. We successfully identified and constructed a satisfactory signature composed of five glycolytic genes and verified it in the validation set. Furthermore, the molecular roles of the signature components in regulating malignant tumor behaviors were validated *in vitro*.

## Materials and Methods

### Collection of Prostate Cancer Data

PCa gene expression data (RNA-Seq) and the corresponding clinical data were downloaded from the TCGA database (https://portal.gdc.cancer.gov) and Gene Expression Omnibus (GEO: https://www.ncbi.nlm.nih.gov/geo/). With |log_2_(fold change)| >0.5 and false discovery rate (FDR) <0.05 as the standards (22), the data were standardized with the “edgeR” package of R software (version 3.6.3, https://www.r-project.org), and then differential expression analysis was performed. The glycolysis-related gene dataset was downloaded from GSEA (http://www.hmdb.ca). The same standard was used to analyze the differentially expressed glycolysis-related genes (DGRGs) between PCa and normal tissues. The relationships between pairs of genes were analyzed through GEPIA (http://gepia.cancer-pku.cn/).

### Construction of a Risk-Scoring Model

Based on the DGRGs obtained from the above screening, univariate Cox regression analysis and LASSO regression analysis were used to screen the prognostic glycolysis-related genes. Then, a separate survival analysis was conducted for each gene to obtain DGRGs with statistically significant associations with survival. Based on the obtained prognosis-related DGRGs, LASSO regression analysis was used to calculate the risk coefficient (coefi) of each DGRG. Then, the sum of the product of the coefi values of all prognostic-related DGRGs and their expression (expri) values for each sample was calculated as the patient risk score $\left(\text{risk score}=\Sigma_{i=1}^{n}({\text{Coef}}_{i}\times {\text{Expr}}_{i})\right)$ (23). Using the median risk score as the cutoff point, patients were divided into high-risk and low-risk groups. Kaplan–Meier survival analysis was used to determine whether disease-free survival (DFS) was significantly different between high-risk and low-risk patients. Receiver operating characteristic (ROC) curve analysis was used to calculate the area under the curve (AUC) to evaluate the predictive performance of the risk-scoring model. Univariate Cox regression and multivariate Cox regression analyses were used to clarify the relationship between the risk score, age, TNM stage, Gleason score, and prognosis of PCa patients.

### External Verification of the Risk-Scoring Model

The GEO dataset GSE70770 was used to verify the risk score model. Similar to the previous approach, the sum of the product of the coefi values of all prognostic-related DGRGs and their expression (expri) values for each sample was calculated as the patient risk score. Kaplan–Meier survival analysis was used to determine whether DFS was significantly different between high-risk and low-risk patients. ROC curve analysis was used to calculate the AUC to evaluate the predictive performance of the risk-scoring model.

### Feature Set Enrichment Analysis

Gene ontology (GO) is widely used in the field of molecular biology and can effectively identify the biological attributes of samples according to high-throughput genetic data. Kyoto Encyclopedia of Genes and Genomes (KEGG) is a collection of high-throughput biological datasets related to genomes, cells, diseases, and signaling pathways. It is usually used to annotate a list of genes and signaling pathways related to a phenotype of interest. The R language package was used to perform GO function and KEGG enrichment analysis of DGRGs.

### Cell Lines and Cell Culture

The human PCa cell line PC3 was purchased from the American Type Culture Collection (ATCC, VA, USA). PC3 cells were cultured in RPMI-1640 (Gibco, NY, USA) medium with 10% fetal bovine serum (FBS, BI, Israel), 100 U/ml penicillin, and 100 μg/ml streptomycin. The cells were cultured in an incubator with 5% CO _2_ at 37°C.

### RNA Interference

Small interfering RNA (siRNA) duplexes targeting the human HMMR, KIF20A, GPR87, PGM2L1, and ANKZF1 genes were synthesized and purified by GenePharma (Suzhou, China). PC3 cells (1 × 10^5^) were seeded in six-well plates and cultured to 50%–70% confluence, and transient transfection was performed with Lipofectamine iMAX (Invitrogen, USA) according to the manufacturer’s instructions. The validated oligo sequences of siRNAs are listed in **Supplementary Table S1**. The detailed procedure is available in **Supplementary File 1**.

### Glycose Consumption and Lactic Acid Production

PC3 cells were digested after transient transfection with siRNA for 48 h, counted (1 × 10^5^), seeded in 12-well plates, and cultured until the cells adhered. The medium was replaced with fresh medium and the initial glucose and lactic acid concentrations were detected. After culturing for 24 h, the culture medium was collected and centrifuged for 15 min at 12,000 rpm at 4°C. A lactate assay kit (Nanjing, China) was used to measure the lactate concentration following the manufacturer’s instructions. The glucose concentration was tested with a glycose assay kit (Nanjing, China) according to the manufacturer’s instructions. Glucose consumption and lactate production within 24 h were calculated according to the difference between the initial and final concentrations according to a standard curve. The detailed procedure is available in **Supplementary File 1**.

### Transwell Assay

Cells were harvested after transient transfection with siRNA for 48 h, and chambers (8-μm pore size, Costar) with or without Matrigel (BD Science, USA) were used for cell invasion assays or migration assays. Approximately 4 × 10^4^ cells (for the migration assay) or 6 × 10^4^ cells (for the invasion assay) were resuspended in 200 μl of serum-free medium and added to the upper chambers. A total of 600 μl of medium containing 10% FBS was placed into the lower chambers. After incubation for 24 h, the cells in the upper chamber were gently removed with cotton swabs, and cells on the lower surface were fixed with paraformaldehyde and stained with 0.1% crystal violet for 20 min at room temperature. Photographs were taken with a microscope (Olympus, Tokyo, Japan), and the cells were counted for analysis.

### Statistical Analysis

All statistical analyses were accomplished with R software (packages: limma, GSVA, GSEABase, sparcl, pheatmap, estimate, ggpubr, e1071, preprocessCore, survival, glmnet, survminer, survivalROC, rms, foreign, timeROC, and ggplot2) and GraphPad Prism (version 7.03). The correlation was determined by Pearson correlation analysis. Chi-square tests and *t*-tests were utilized to compare clinical variables. Survival status was assessed by Cox regression analysis. DFS curves were generated by the Kaplan–Meier method and evaluated by the log-rank test. A two-tailed *p* < 0.05 was considered to indicate statistical significance.

## Results

### Initial Screening and Identification of Differentially Expressed Glycolysis-Related Genes

**Figure 1:**
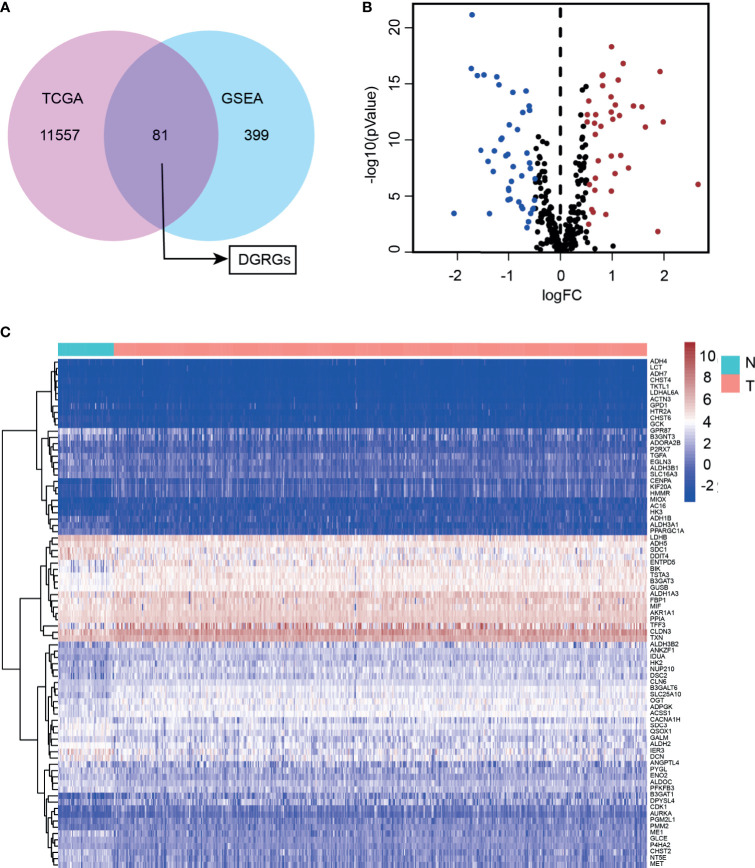
Identification of differentially expressed glycolysis-related genes in prostate cancer cohort. **(A)** Venn diagram showed overlapping of TCGA and GEO70770 database. **(B)** Volcano plot of differentially expressed glycolysis-associated genes expressed mRNA. **(C)** Heatmap of the differentially expressed glycolysis-associated genes showing expression signature in patients with PCa.

Transcriptome sequencing data and detailed clinical follow-up information derived from 498 PCa patients were downloaded from the TCGA. With screening thresholds of |log_2_(fold change)|>0.5 and FDR<0.05, the DGRG profile was generated through the “edgeR” R package (**Supplementary Figures S1A, B**). Then, five glycolysis or glycolysis pathway-related gene sets were obtained from GSEA (**Supplementary Table S2**). By analyzing the overlaps between the differential transcriptome expression profile and GSEA gene sets, DGRGs were identified (**Figure 1A**). Volcano and heatmaps showed the expression patterns of the DGRGs (**Figures 1B, C**).

### Identifying Candidate Molecules Associated With the Prognosis of Prostate Cancer Patients

**Figure 2:**
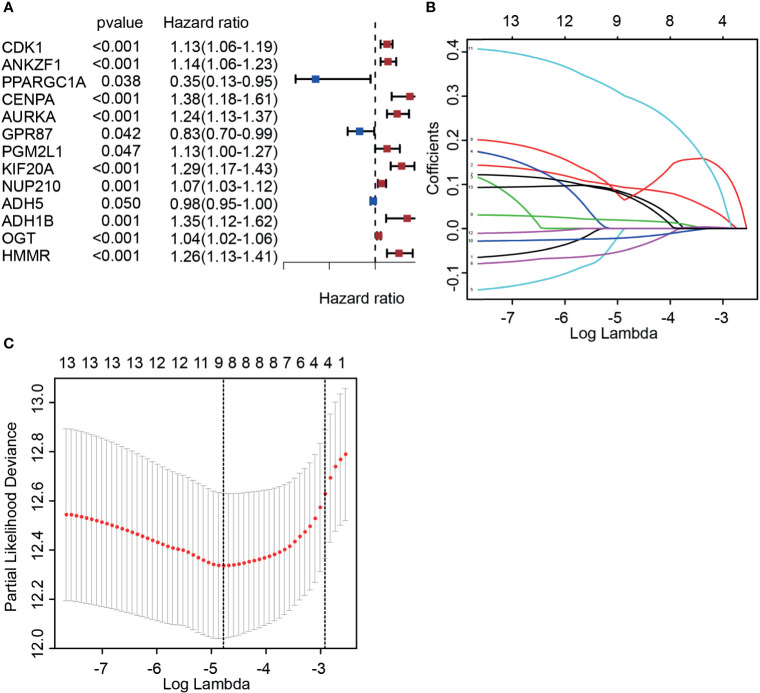
Identifying candidate molecules associated with the prognosis of prostate cancer patients. **(A)** Univariate Cox regression analysis of glycolysis-associated genes. **(B, C)** Determination of the best penalty value (−5 < λ < −4). LASSO regression of screening glycolysis-associated genes with patients’ prognosis.

To fully understand the role of DGRGs in PCa patients, univariate Cox regression was carried out to preliminarily analyze this profile. The results indicated that 13 genes were significantly associated with the prognosis of PCa patients (**Figure 2A**). Considering the possibility of overfitting that affects the authenticity of the results, LASSO regression was applied to further screen glycolysis-related genes associated with patient prognosis among these 13 genes. The data showed that after optimization of penalty parameters, eight genes (HMMR, KIF20A, PGM2L1, ANKZF1, GPR87, ADH5, ADH1B, NUP210) were confirmed to be related to the prognosis of PCa patients (**Figures 2B, C**).

### Validation of the Expression and Prognosis of Candidate Molecules

**Figure 3:**
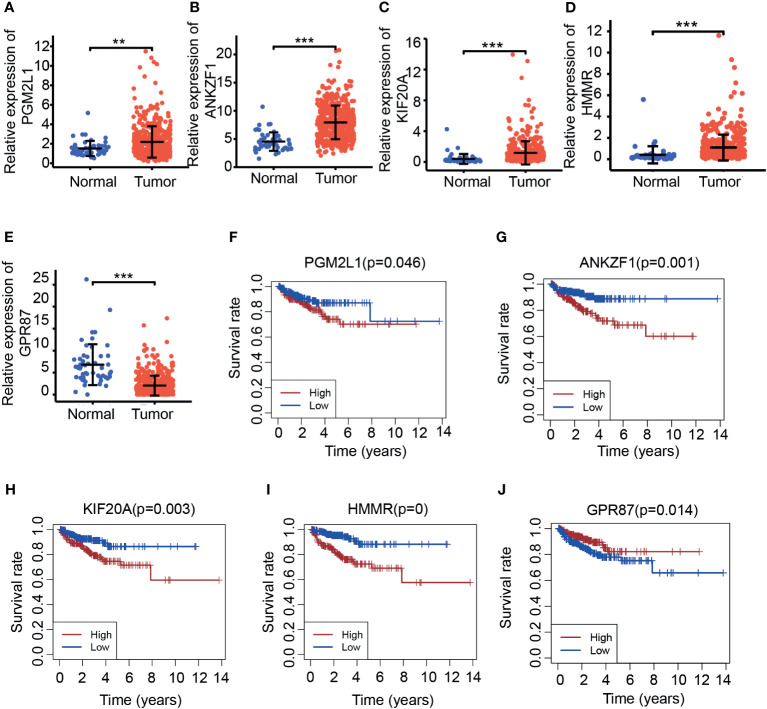
Investigation of the expression of candidate molecules based on TCGA. **(A–E).** Expression of PGM2L1 **(A)**, ANKZF1 **(B)**, KIF20A **(C)**, HMMR **(D)**, and GPR87 **(E)** in TCGA. **p < 0.01, ***p < 0.001. **(F–J)**. Disease-free survival of PGM2L1 **(F)**, ANKZF1 **(G)**, KIF20A **(H)**, HMMR **(I)**, and GPR87 **(J)** in TCGA.

We next aimed to further confirm the possible roles of these eight genes in PCa. Based on the PCa patient cohort derived from TCGA datasets, the expression and predictive ability of the eight glycolysis-related genes were tested. Consistent with previous results, the expression of four glycolysis-related genes (PGM2L1, ANKZF, KIF20A, and HMMR) was upregulated in cancer tissues compared with normal tissues (**Figures 3A–D**), and analysis of the DFS rate showed that high expression of these genes predicted a poor prognosis in the cancer group (*p* < 0.05) (**Figures 3F–I**). In addition, PCa patients with high expression of GPR87 had a longer survival time (**Figures 3E, J**), which indicated that it may act as a tumor suppressor. However, three glycolysis-related genes (ADH1B, ADH5, and NUP210) were significantly differentially expressed at the mRNA level in tumor tissues versus adjacent normal tissues (**Supplementary Figures S2A–C**) but were not significantly related to patient prognosis (*p* > 0.05) (**Supplementary Figures S2D–F**). Taking all the results into account, five DGRGs (HMMR, KIF20A, GPR87, PGM2L1, and ANKZF1) were subsequently used to construct a prediction model for PCa patients.

### Construction of a Risk Score Model Based on Differentially Expressed Glycolysis-Related Genes

**Figure 4:**
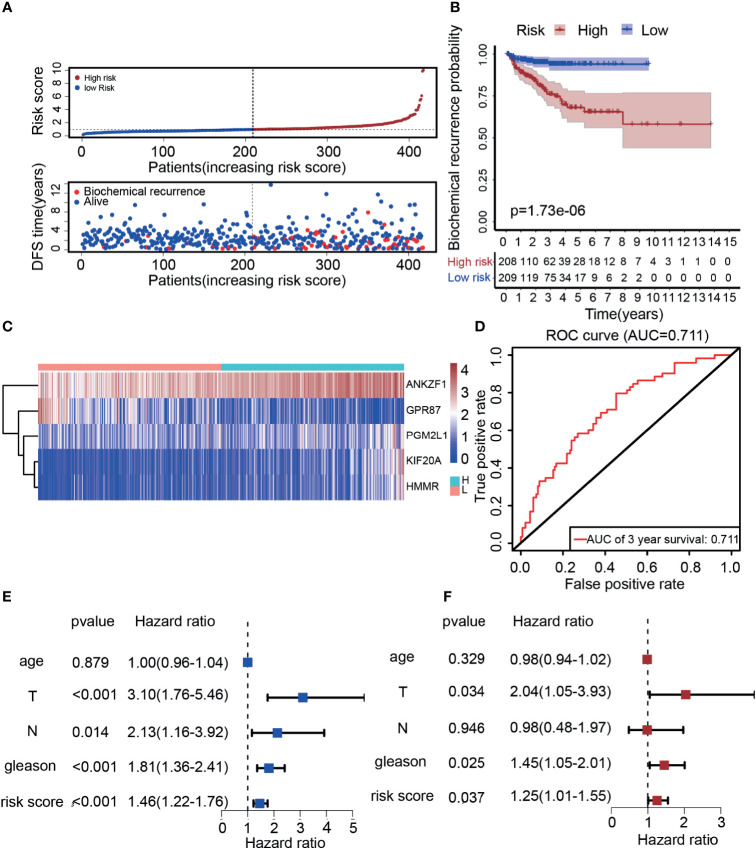
Construction of a risk score model based on five glycolysis-related genes. **(A)** The distribution of the five-mRNA risk score and survival status for each patient. **(B)** Kaplan–Meier curve of survival time in high-risk and low-risk group. **(C)** Expression of five glycolytic genes in the high-risk group and low-risk group based on TCGA. **(D)** ROC curves of the glycolysis-related signature for prediction of DFS of patients with PCa (AUC = 0.711). **(E, F)** Univariate and multivariate Cox regression analysis of clinical information (Age, T stage, N stage, Gleason score, and risk score).

According to previous results, five genes were ultimately selected for the construction of the risk score model. Based on the expression (Expr) and coefficient value of each gene (**Table 1**), a prognostic model for predicting the survival time of each patient was developed as follows: $\text{risk score}=\Sigma_{i=1}^{n}(\text{Coe}{\text{f}}_{i}\times \text{Exp}{\text{r}}_{i})$. On the basis of the result for each patient, PCa patients in the TCGA cohort were divided into two groups (high-risk group and low-risk group) according to the median value. As expected, there were more deaths in the high-risk group than in the low-risk group (**Figure 4A**). This result implied that patients in the low-risk group had a better prognosis than those in the high-risk group, indicating the excellent predictive effect of this model. Next, to further confirm this result, DFS analysis was performed, and the Kaplan–Meier survival curves clearly showed that patients with a high-risk score had a poorer clinical outcome than those with a low-risk score (**Figure 4B**). In addition, we analyzed the five-gene signature in the high-risk and low-risk groups. As presented in **Figure 4C**, ANKZF1, PGM2L1, KIF20A, and HMMR were significantly upregulated in the high-risk group, and GPR87 was downregulated in the high-risk group. The time-dependent ROC curve showed that the AUC was 0.711 (**Figure 4D**). Some clinical factors, such as age, TNM stage, and Gleason score, are important factors for judging the prognosis of PCa. Thus, we explored whether the Gleason score, T stage, N stage, and risk score were independent predictors of PCa prognosis. Univariate and multivariate Cox regression analyses were carried out. The results demonstrated that T stage, Gleason score, and risk score were independent predictors of prognosis in patients with PCa (**Figures 4E, F**). Taken together, these findings showed that the risk score based on the signature of five glycolysis-related genes was an independent predictor of prognosis in patients with PCa.

**Table 1:** The detailed information of five glycolysis-related genes for the prediction model.

Gene	Coef	HR (95% CI)	*p*-value
HMMR	0.0714607323453938	1.26 (1.13–1.41)	<0.0001
KIF20A	0.0697796872729206	1.29 (1.17–1.43)	<0.0001
GPR87	−0.0467478802686722	0.83 (0.70–0.99)	0.041788994
PGM2L1	0.0775653229914858	1.12 (1.00–1.27)	0.046947454
ANKZF1	0.0919194115211566	1.14 (1.06–1.23)	0.000321331

$\text{risk score}=\Sigma_{i=1}^{n}(\text{Coe}{\text{f}}_{i}\times \text{Exp}{\text{r}}_{i})$

### External Dataset to Verify the Predictive Performance of the Risk Score Model

**Figure 5:**
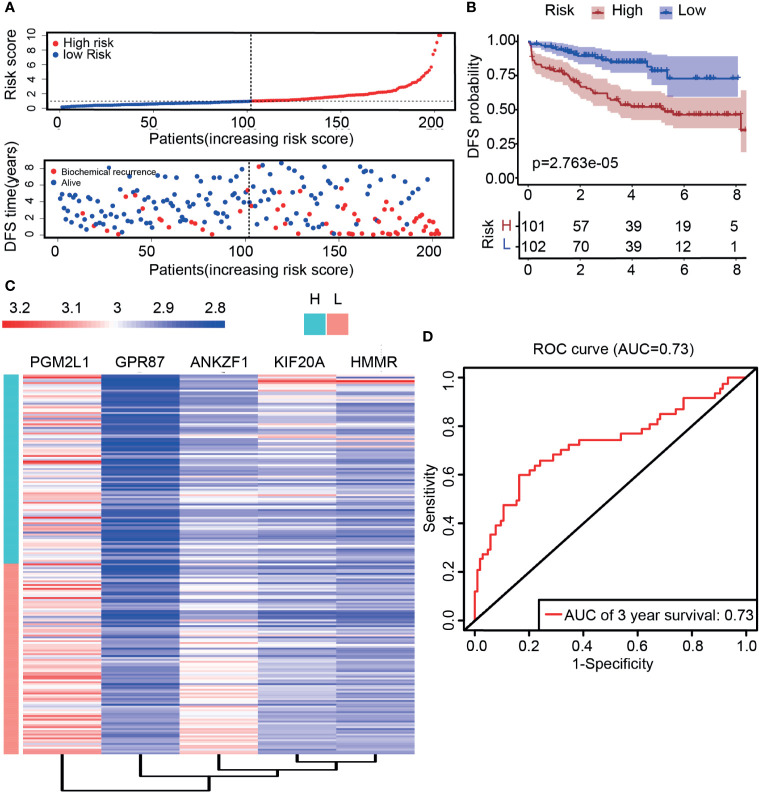
Validation of risk score through external data derived from the GEO dataset (GSE70770). **(A)** The distribution of the five-mRNA risk score and survival status for each patient. **(B)** Kaplan–Meier curve of survival time in high- and low-risk groups. **(C)** Expression of five glycolytic genes in the high-risk group and low-risk group based on GSE70770. **(D)** ROC curve of DFS (AUC = 0.73) in validation datasets.

Previous data showed that the prediction model had a better predictive performance in the TCGA cohort, but it was unclear whether the prediction model has the ability to judge prognosis precisely in other cohorts. To this end, the GSE70770 dataset derived from the GEO was used to verify the predictive performance. The expression of these five genes was analyzed, and the results showed that HMMR, KIF20A, and ANKZF1 were upregulated in cancer tissues, while GPR87 was downregulated in cancer tissues, which were consistent with previous results (**Supplementary Figures S3A–D**). However, the expression of ANKZF1 showed no significant difference in cancer and normal tissues (**Supplementary Figure S3E**). Similarly, patients with PCa were classified into a high-risk group and a low-risk group according to the five-gene signature score for each patient. The mortality rate was notably increased in the high-risk group, suggesting that patients in the high-risk group had a poorer prognosis than those in the low-risk group (**Figure 5A**). DFS data also showed that the survival time was shorter in patients with high-risk scores than those with low-risk scores (**Figure 5B**). In addition, in the high-risk group, the expression of ANKZF1, PGM2L1, KIF20A, and HMMR were increased remarkably, while GPR87 was downregulated (**Figure 5C**). Based on the ROC curve, the AUC was 0.73, suggesting good predictive ability (**Figure 5D**).

### Molecular Functions of the Five Genes in Glycose Consumption and Lactic Acid Production

**Figure 6:**
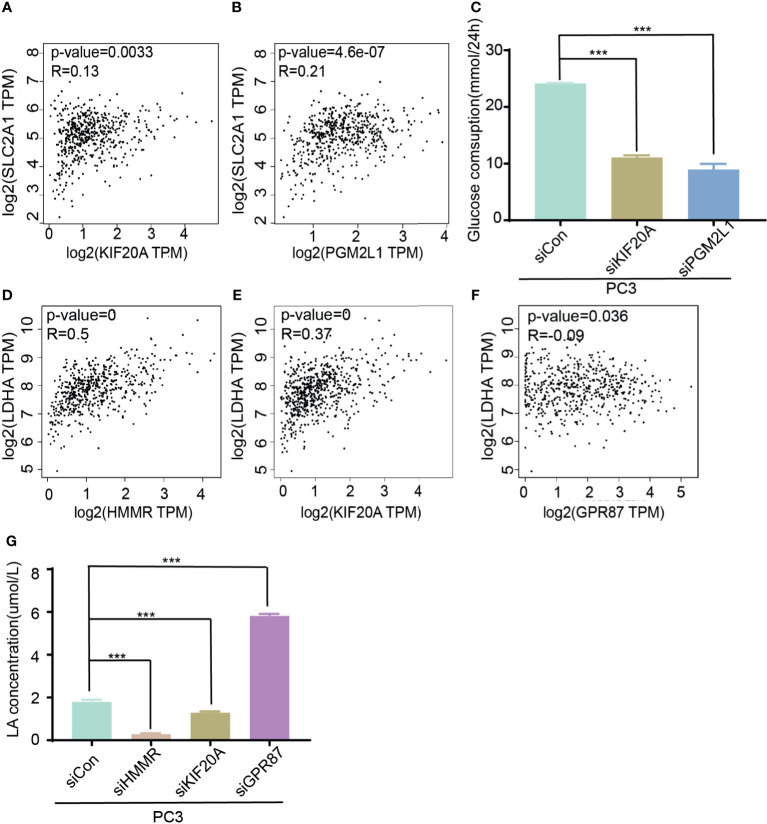
Molecular function of five genes in glycose consumption and lactic acid production. **(A, B)** Analysis of corrections of SLC2A1 with KIF20A **(A)** or PGM2L1 **(B)**. **(C)** Detection of glucose consumption in control or siRNA (KIF20A and PGM2L1) group. ***p < 0.001. **(D–F)** Analysis of correction of LDHA with HMMR **(D)**, KIF20A **(E)**, and GPR87 **(F)**. **(G)** Investigation of lactic production in control and siRNA (HMMR, KIF20A, and GPR87) group. ***p < 0.001.

Considering that these genes are derived from glycolytic gene sets, they are likely to exert biological functions through changing the metabolic pattern of tumor cells. Hence, glycose consumption assays and lactic acid production assays were subsequently adopted. SLC2A1, also known as glucose transporter Glut-1, is the vital factor for the first step of glucose uptake, and LDHA is the rate-limiting enzyme that catalyzes the production of lactic acid. They are both vital regulators involved in regulating development of malignancy (24, 25). Therefore, to validate the roles of these five genes in glucose metabolism, the correlations among the five genes and SLC2A1 or LDHA were assessed. The data showed that HMMR, GPR87, KIF20A, and PGM2L1 were positively correlated with SLC2A1 and that ANKZF1 was negatively correlated with SLC2A1 (**Figures 6A, B** and **Supplementary Figures S4A–C**). Similarly, HMMR, KIF20A, PGM2L1, and ANKZF1 were positively correlated with LDHA, whereas GPR87 had a negative correlation with LDHA (**Figures 6D–F** and **Supplementary Figures S4D, E**). To confirm the results, glucose consumption and lactic acid production were assessed after gene knockdown and knockdown efficiency confirmation (**Supplementary Figures S5A–E**); the results demonstrated that glucose consumption decreased markedly when KIF20A or PGM2L1 was knocked down (**Figure 6C**), while knockdown of HMMR, GPR87, and ANKZF1 did not induce a significant change (**Supplementary Figure S6A**). The lactic acid production data showed that the concentration of lactic acid increased when GPR87 expression decreased but markedly decreased after knockdown of KIF20A or HMMR (**Figure 6G**); furthermore, there were no significant changes with PGM2L1 or ANKZF1 knockdown (**Supplementary Figure S6B**). In summary, the glycolysis-related signature truly impacted the glycolysis process in PCa cells.

### Effect of the Five Glycolytic Genes on the Migration and Invasion of Prostate Cancer Cells

**Figure 7:**
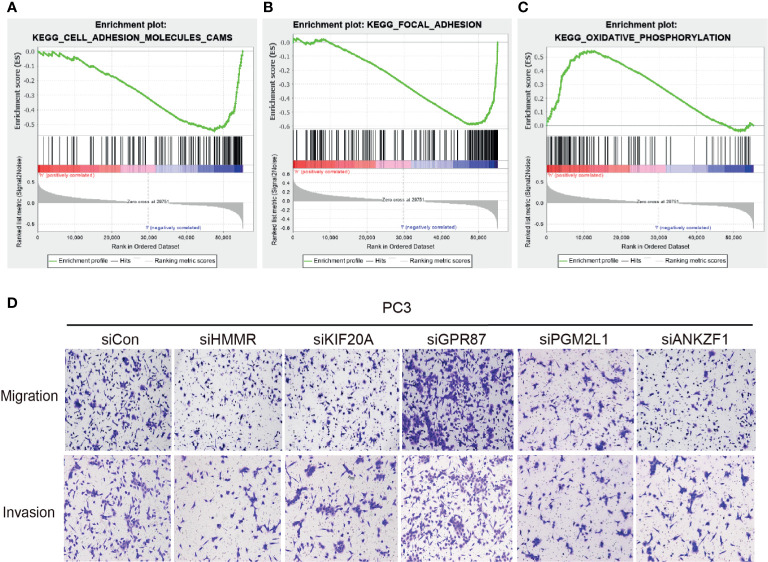
Effect of the five glycolytic genes on the migration and invasion of prostate cancer cells. **(A–C)** GESA analysis was performed in the high-risk group and low-risk group. **(D)** Investigation of ability of migration and invasion of PC3 cells.

After confirming the involvement of the glycolysis-related signature in the glucose metabolism process, we further explored whether they affect the progression of PCa. Single-sample GSEA was used to explore the possible molecular functions and signaling pathways that might be involved. The results indicated that metastasis-associated pathways (cell adhesion molecules [CAMs], focal adhesion) and oxidative phosphorylation pathways were significantly enriched among these genes (**Figures 7A–C**). Considering these results, the migration and invasion ability of PC3 cells were assessed after knocking down different target genes. The results clearly demonstrated that knockdown of HMMR, KIF20A, PGM2L1, or ANKZF1 significantly attenuated the invasion and migration of PC3 cells. However, GPR87 knockdown promoted the invasion and migration of PC3 cells (**Figure 7D**). After confirmation *in vitro*, it was clear that this glycolysis-related signature exerts important regulatory effects in the progression of PCa.

## Discussion

Although improvements in treatment have greatly increased the survival of PCa patients (26–29), survival and quality of life deteriorate significantly once BCR occurs (30). More regrettably, it is unavoidable that approximately 50% of high-risk PCa patients experience BCR despite active treatment (31). Moreover, the genome sequencing results shows that there are great individual differences in PCa, indicating the heterogeneity of PCa. These results pose great challenges in the formulation of favorable and reasonable personalized treatment strategies (32). Given that metabolic reprogramming is a crucial hallmark of malignancy, several studies have successfully recognized glycolysis-associated factors in multiple solid tumors (13, 33–35). Hence, we constructed and verified a prediction model based on glycolysis-related genes to accurately judge the prognosis of PCa patients.

Initially, in order to obtain sufficient meaningful variables, differentially expressed genes in cancer tissues were analyzed in the TCGA and subsequently overlapped with glycolysis-associated gene sets downloaded from the GSEA database. In addition to univariate and multivariate regression analysis, LASSO is another scientific variable screening method that is widely used to construct prediction tools (36). With these models, we further screened 13 glycolysis-related genes associated with prognosis. Considering that inclusion of many variates in signature could reduce the significance of research and hinder further application, we analyzed their expression pattern and HR values; five glycolytic genes (HMMR, KIF20A, GPR87, ANKZF1, and PGM2L1) were eventually selected for further investigation. ROC curve analysis confirmed the effectiveness of this model, which indicated that patients with risk scores had a poor prognosis. In addition, a validation dataset (GSE70770) was used to evaluate the predictive performance of the model in the present study, which makes the tool more valuable. Probably due to the different quality control of each batch of sequencing, the expression of these five genes was not completely consistent with TCGA. Even so, we found that the model still had a good predictive performance in PCa with BCR. However, due to the lack of a large cohort for clinical validation, the actual value of this prediction model still needs to be further evaluated in the clinic.

Increasing evidences have proven that the excessive activation of glycolysis mediates cell proliferation, resistance to chemotherapy, the immune response, and distant metastasis (15, 37–39), so targeting aerobic glycolysis has become a promising treatment strategy (40). In the present study, the molecular functions of five glycolytic genes were investigated *in vitro*. Previous literature clearly indicates that HMMR and KIF20A function as oncogenes in many types of cancers. For example, activation of the TGF-β/Smad2 signaling pathway mediated by HMMR contributes to the chemoresistance of gastric cancer (41). KIF20A maintains a set of malignant characteristics in colorectal cancer by activating the JAK/STAT3 pathway (42, 43). However, little is known about GPR87, PGM2L1, and ANKZF1 in PCa. In the aspect of BCR, few studies have reported these glycolytic genes’ functions in the process of BCR. For patients who underwent radical prostatectomy (RP), BCR was a precursor to local tumor recurrence and distant metastasis (44). The regulatory roles of these glycolytic genes in the process of metastasis were assessed, which was expected to partially reflect their roles in the process of BCR. In the present study, the results of the Transwell assays of HMMR and KIF20A knockdown cells further confirmed their roles as oncogenes in PCa, which were consistent with previous studies. Furthermore, this is the first study to prove the anti-oncogenic or oncogenic role of GPR87, PGM2L1, and ANKZF1 in PCa progression, providing important information for further investigating PCa. Interestingly, it is worth noting that GPR87 is generally considered as a tumor driver in the tumorigenesis of pancreatic cancer (45), lung cancer (46), bladder cancer (47), and liver cancer (48). However, it was indicated as a tumor suppressor in PCa. GPR87 is widely known as a G protein-coupled receptor with seven transmembrane proteins, and the signaling pathways that it activates are substrate-dependent in terms of mechanism. For instance, GPR87 couples with Gα_q_ to mediate the activation of CREB and NF-κB signaling pathway and couples with Gα_12/13_ to induce SRE activation (49, 50). The specific characteristics of GPR87 in PCa and the detailed mechanisms are unclear and deserve further investigation.

In addition, several bioinformatics analyses have reported that these genes (HMMR, KIF20A, PGM2L1, ANKZF1, and GPR87) participate in glycolysis (51, 52). However, their roles in regulating tumor glycolysis have rarely been confirmed. Therefore, it was unclear whether they participated in regulating glucose metabolism in cancer cells. In the present study, we not only confirmed their involvement in the glycolysis process of PCa through bioinformatics but also demonstrated that interfering with KIF20A and PGM2L1 expression could affect glucose consumption and that interfering with HMMR, KIF20A, and GPR87 expression could regulate lactic acid production *in vitro*. It was worth noting that ANKZF1 had little regulatory role in glucose metabolism and lactic acid production process. It seems that the main function of ANKZF1 was to participate in the process of ribosome biogenesis (53, 54). Due to the lack of sufficient research, the actual role of ANKZF1 in cancer and glycolysis is unclear, and it is worthy of further investigation. In conclusion, this study preliminarily revealed their roles in the regulation of glucose metabolism.

Overall, further functional verification *in vitro* not only made our results more comprehensive but also provided a theoretical basis for this model. However, little is known about the exact roles of these genes and corresponding signaling pathways in regulating PCa progression and glycolysis. To better understand the theoretical basis of this model, the biological roles of these genes *in vivo* and their corresponding precise molecular mechanisms should be further elucidated in the future.

## Conclusion

By analyzing the transcriptome signature of TCGA and GEO cohorts, a risk score system based on five glycolysis-associated genes was constructed and exhibited excellent performance in judging the prognosis of individual patients. In addition, GSEA and *in vitro* functional assays implied that these genes function through metastasis-related pathways and glucose metabolism pathways, indicating that they truly have a regulatory effect on tumor progression. Our findings indicated that the risk score prediction model might be a potential prognostic predictor for PCa patients with BCR.

## Data Availability Statement

Publicly available datasets were analyzed in this study: The Cancer Genome Atlas (https://portal.gdc.cancer.gov/); the GSE70770 dataset was downloaded from Gene Expression Omnibus (GEO: https://www.ncbi.nlm.nih.gov/geo/).

## Ethics Statement

The studies involving human participants were reviewed and approved by the Ethical Review Committee of Sun Yat-sen Memorial Hospital of Sun Yat-sen University. Written informed consent for participation was not required for this study in accordance with the national legislation and the institutional requirements.

## Author Contributions

KX, KL, KG, and CoL conceived and designed the study. CoL performed public database data collection and analysis. KG and JS performed the experiments and analyzed the data. KG wrote the manuscript. ChL, ZT, and JS contributed to manuscript revision. All authors contributed to the article and approved the submitted version.

## Funding

This work was supported by the National Natural Science Foundation of China (Grant Nos. 81702528, 81702525, and 82072841), the Guangdong Basic and Applied Basic Research Foundation (2021A1515010199), the Science and Technology Program of Guangzhou (Grant No. 201803010029), the Medical Scientific Research Foundation of Guangdong Province (Grant No. C2018060), and the Yixian Clinical Research Project of Sun Yat-sen Memorial Hospital (Grant No. SYS-C-201802).

## Conflict of Interest

The authors declare that the research was conducted in the absence of any commercial or financial relationships that could be construed as a potential conflict of interest.

## Publisher’s Note

All claims expressed in this article are solely those of the authors and do not necessarily represent those of their affiliated organizations, or those of the publisher, the editors and the reviewers. Any product that may be evaluated in this article, or claim that may be made by its manufacturer, is not guaranteed or endorsed by the publisher.

## Supplementary Material

Supplementary Figure S1 Identification of differential expressed genes in TCGA. **(A)** Heatmap of the differential expressed genes in TCGA. **(B)** Volcano plot of the differential expressed genes in TCGA.

Supplementary Figure S2 Expression pattern and DFS of glycolysis-related genes in TCGA. **(A–C)** Expression pattern of ADH1B **(A)**, ADH5 **(B)**, NUP210 **(C)** in tumor tissues and normal tissues. **(D-F)** DFS of ADH1B **(D)**, ADH5 **(E)**, NUP210 **(F)** in prostate cancer patients.

Supplementary Figure S3 Expression of candidate five glycolysis-related genes based on external dataset (GSE70770). **(A-E)**. Expression of HMMR **(A)**, KIF20A **(B)**, ANKZF1 **(C)**, GPR87 **(D)**, PGM2L1 **(E)** in cancer tissues and normal tissues

Supplementary Figure S4 Relationship between glycolysis-related genes with SLC2A1 or LDHA. **(A-C)** Relationship between SLC2A1 with HMMR **(A)**, GPR87 **(B)**, ANKZF1**(C)** in TCGA. **(D, E)** Relationship between LDHA with ANKZF1**(D)**, PGM2L1**(E)** in TCGA.

Supplementary Figure S5 Knockdown efficiency confirmation of glycolysis-related genes in prostate cacner cell lines through qRT-PCR. **(A–E)** Expression of GPR87**(A)**, ANKZF1**(B)**, HMMR**(C)**, PGM2L1**(D)**, KIF20A**(E)** in prostate cancer cells.

Supplementary Figure S6 Glucose consumption and lactic acid production in glycolysis-related genes. **(A)** Glucose consumption in HMMR, ANKZF1, GPR87 knock down group. **(B)** Lactic acid production in PGM2L1, ANKZF1 knock down group.
