# Supplementary figures and images for: A Novel Risk Factor Model Based on Glycolysis-Associated Genes for Predicting the Prognosis of Patients With Prostate Cancer
Source: Front Oncol. 2021 Sep 14;11:605810. doi: 10.3389/fonc.2021.605810 (PMC8476926; doi:10.3389/fonc.2021.605810)

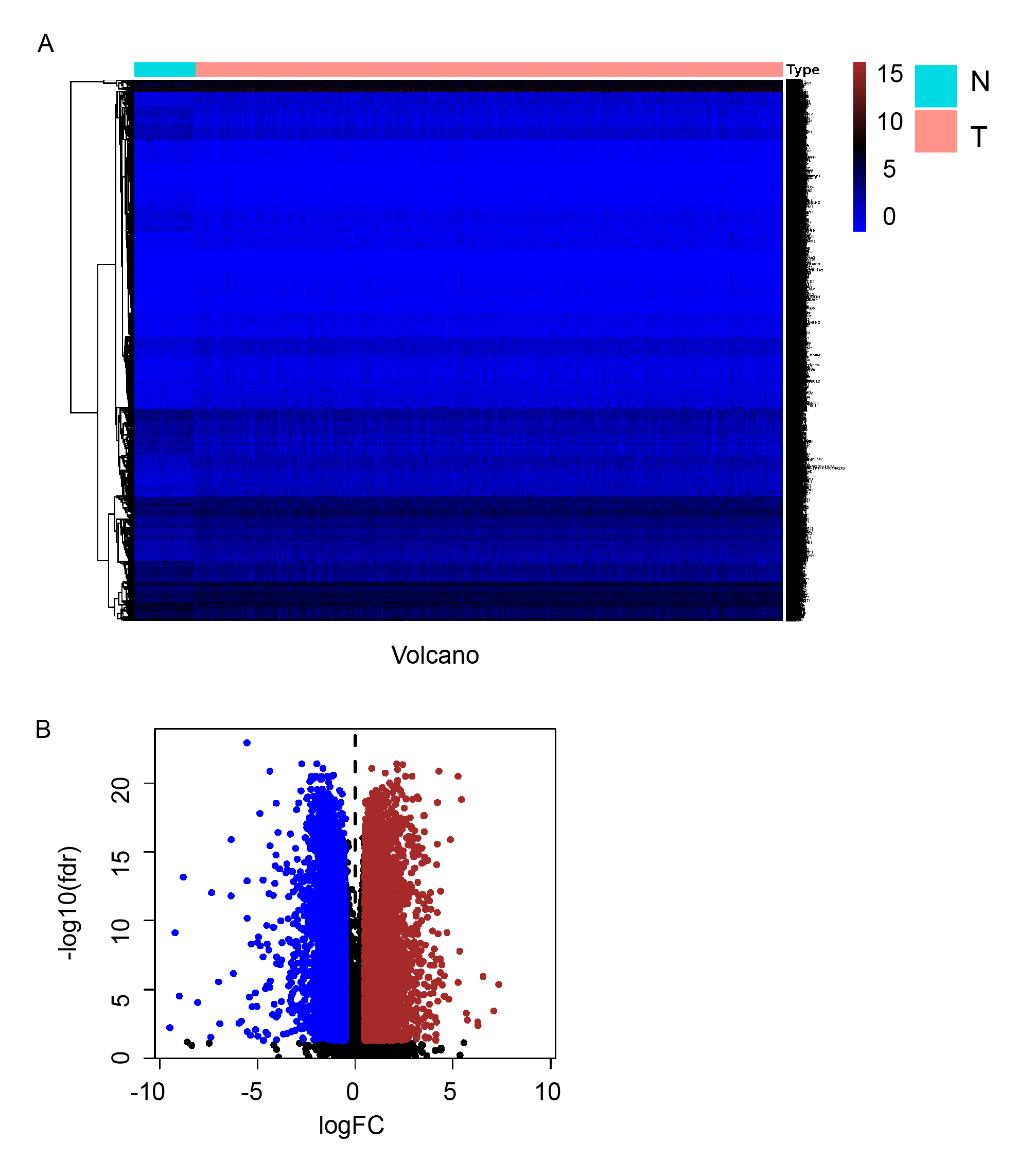

Supplement: Supplementary Figure S1 — Identification of differential expressed genes in TCGA. (A) Heatmap of the differential expressed genes in TCGA. (B) Volcano plot of the differential expressed genes in TCGA. [file Image_1.jpeg]

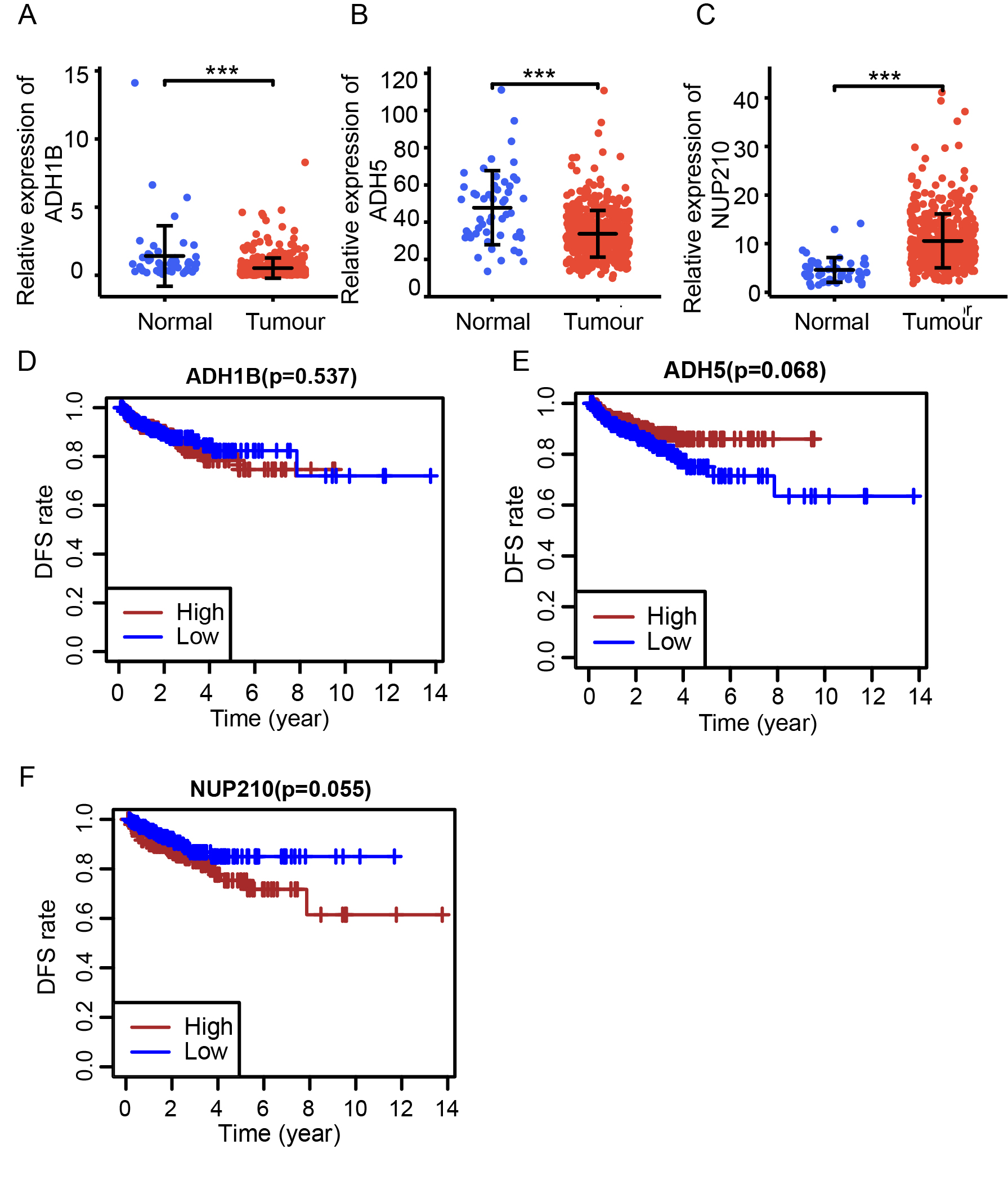

Supplement: Supplementary Figure S2 — Expression pattern and DFS of glycolysis-related genes in TCGA. (A–C) Expression pattern of ADH1B (A), ADH5 (B), NUP210 (C) in tumor tissues and normal tissues. (D-F) DFS of ADH1B (D), ADH5 (E), NUP210 (F) in prostate cancer patients. [file Image_2.jpeg]

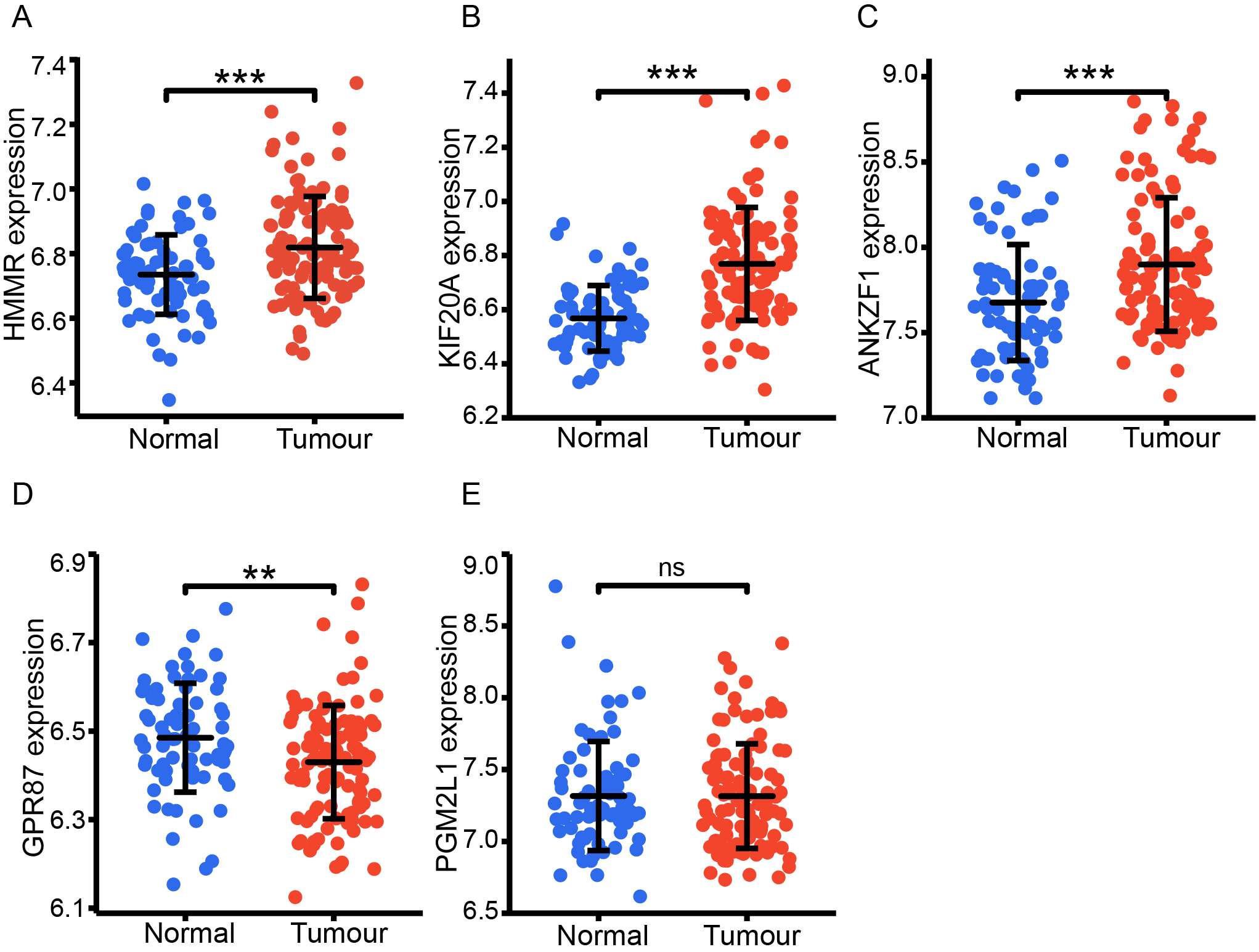

Supplement: Supplementary Figure S3 — Expression of candidate five glycolysis-related genes based on external dataset (GSE70770). (A-E). Expression of HMMR (A), KIF20A (B), ANKZF1 (C), GPR87 (D), PGM2L1 (E) in cancer tissues and normal tissues [file Image_3.jpeg]

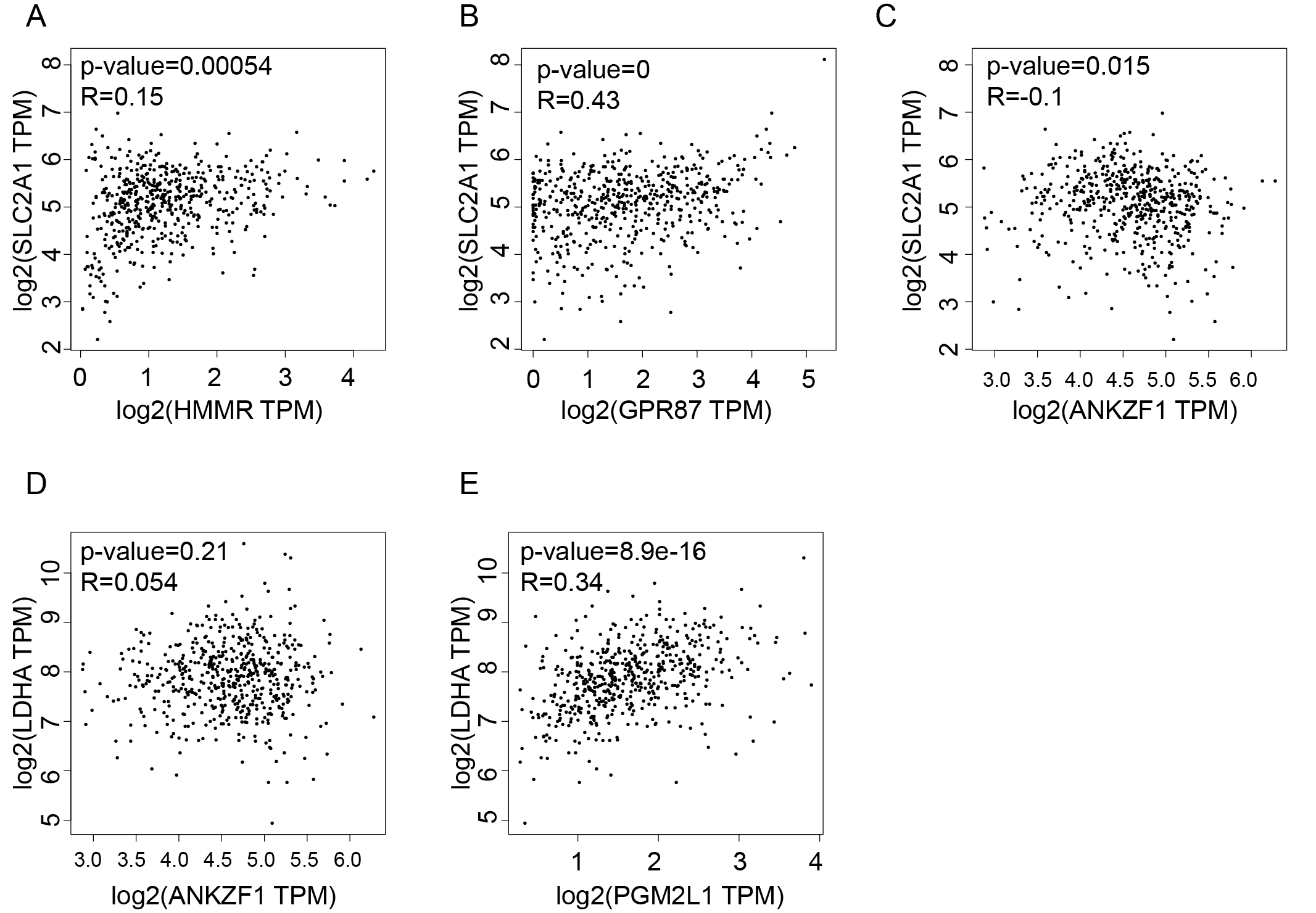

Supplement: Supplementary Figure S4 — Relationship between glycolysis-related genes with SLC2A1 or LDHA. (A-C) Relationship between SLC2A1 with HMMR (A), GPR87 (B), ANKZF1(C) in TCGA. (D, E) Relationship between LDHA with ANKZF1(D), PGM2L1(E) in TCGA. [file Image_4.jpeg]

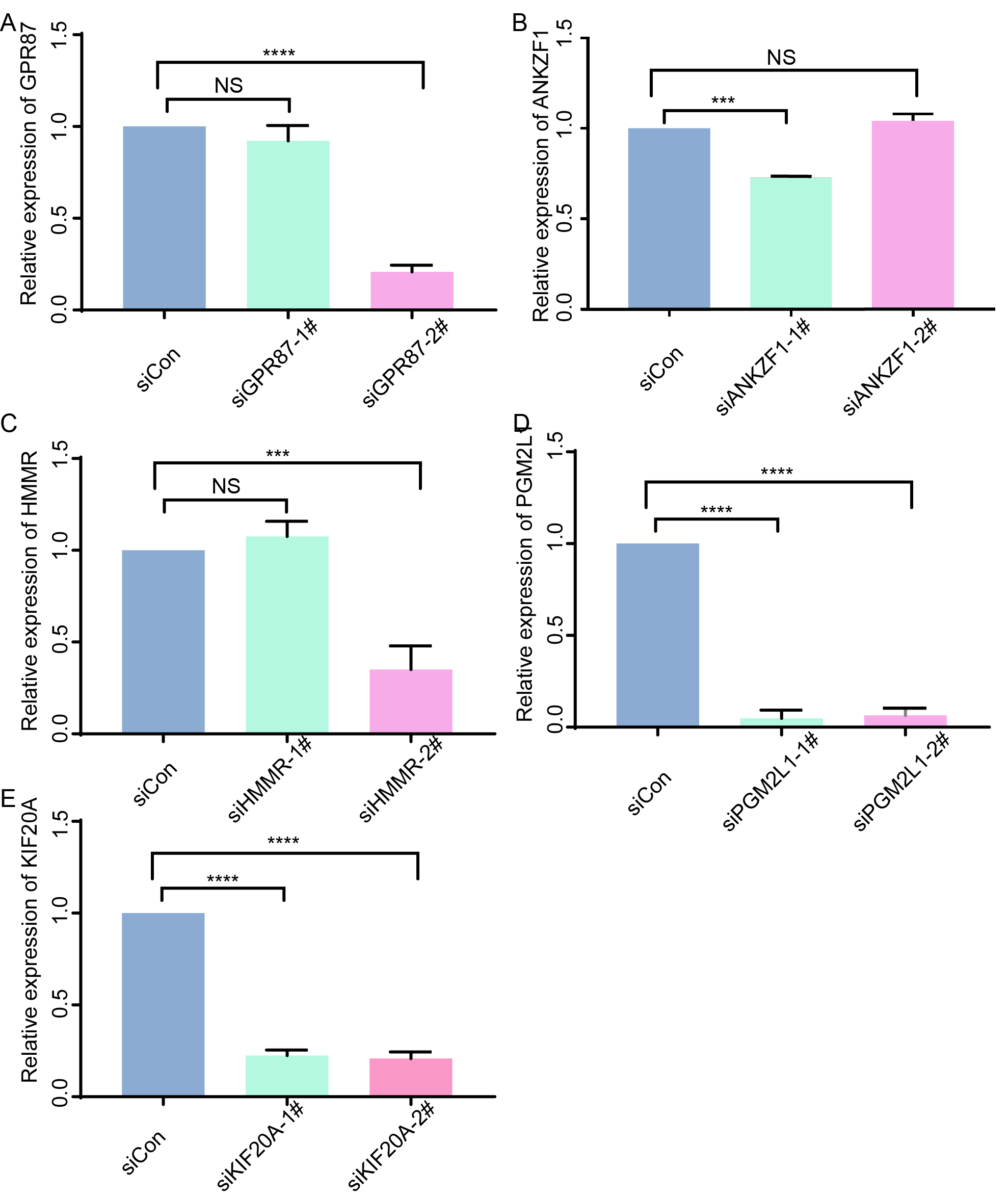

Supplement: Supplementary Figure S5 — Knockdown efficiency confirmation of glycolysis-related genes in prostate cacner cell lines through qRT-PCR. (A–E) Expression of GPR87(A), ANKZF1(B), HMMR(C), PGM2L1(D), KIF20A(E) in prostate cancer cells. [file Image_5.jpeg]

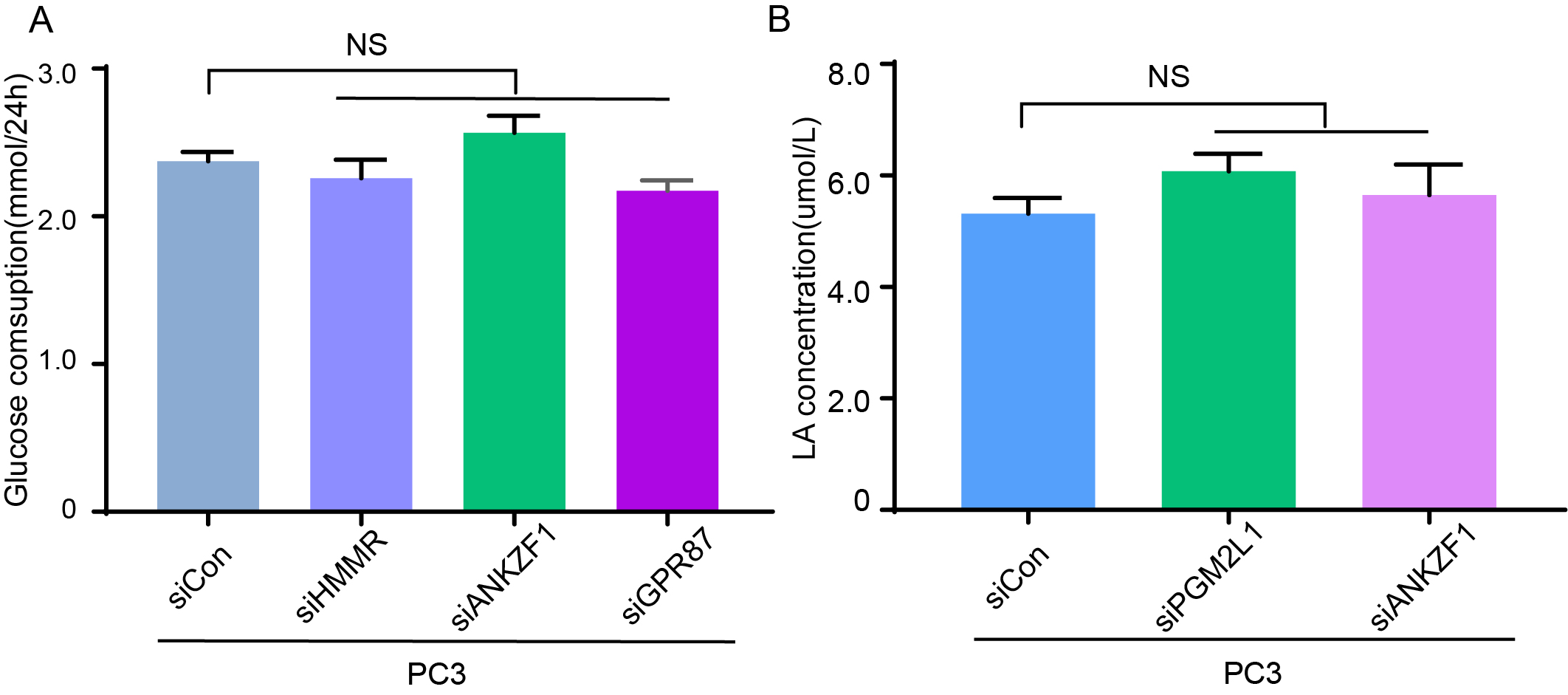

Supplement: Supplementary Figure S6 — Glucose consumption and lactic acid production in glycolysis-related genes. (A) Glucose consumption in HMMR, ANKZF1, GPR87 knock down group. (B) Lactic acid production in PGM2L1, ANKZF1 knock down group. [file Image_6.jpeg]
