# Supplementary material for: A Novel Risk Factor Model Based on Glycolysis-Associated Genes for Predicting the Prognosis of Patients With Prostate Cancer
Source: Front Oncol. 2021 Sep 14;11:605810. doi: 10.3389/fonc.2021.605810 (PMC8476926; doi:10.3389/fonc.2021.605810)
Supplement: Supplementary file 7 [file DataSheet_1.docx]

**RNA interference**

PC3 cells seeded into 6-well plate and culture to 50-70% confluence. Configure transfection solution A: 3ul iMAX dissolved into 250ul Opti-MEM and incubated at room temperature for 5min. Configure transfection solution B: 6ul siRNA solution (Dissolve according to instructions) for target gene or negative control dissolved into 250ul Opti-MEM and incubated at room temperature for 5min. Mixed solution A and solution B and then incubated at room temperature for 20min. Adding the mixture into plate and up to 2ml volume with fresh medium for each plate. After 24 hours, fresh medium was replaced for further cultured and subsequent experiments.

**Glycose consumption and lactic acid production**

For glycose consumption or lactic acid production assay, PC3 cells were counted (1×10^5) and seeded into 12-well plate for ordinary culture. After cultured for 24h, replace with fresh medium and detected the initial glucose and lactic acid concentration as following procedures.

1. Prepare all reagents before starting assay procedure. It is recommended that all Standards and Samples be added in duplicate to the Microelisa Stripplate.

2. Add standard: Set Standard wells, testing sample wells. Add standard 50μl to standard well.

3. Add Sample: Add testing sample 10μl then add Sample Diluent 40μl to testing sample well; Blank well doesn’t add anyting.

4. Add 100μl of HRP-conjugate reagent to each well, cover with an adhesive strip and incubate for 60 minutes at 37°C.

5. Aspirate each well and wash, repeating the process four times for a total of five washes. Wash by filling each well with Wash Solution (400μl) using a squirt bottle, manifold dispenser or autowasher. Complete removal of liquid at each step is essential to good performance. After the last wash, remove any remaining Wash Solution by aspirating or decanting. Invert the plate and blot it against clean paper towels.

6. Add chromogen solution A 50μl and chromogen solution B 50μl to each well. Gently mix and incubate for 15 minutes at 37°C. Protect from light.

7. Add 50μl Stop Solution to each well. The color in the wells should change from blue to yellow. If the color in the wells is green or the color change does not appear uniform, gently tap the plate to ensure thorough mixing.

8. Read the Optical Density at 450 nm using a microtiter plate reader within 15 minutes.
