# Supplementary material for: A Novel Risk Factor Model Based on Glycolysis-Associated Genes for Predicting the Prognosis of Patients With Prostate Cancer
Source: Front Oncol. 2021 Sep 14;11:605810. doi: 10.3389/fonc.2021.605810 (PMC8476926; doi:10.3389/fonc.2021.605810)
Supplement: Supplementary file 8 [file Table_1.docx]

Table S1. Oligo valid sequence of five glycolysis genes

| Genes | Forward (5’-3’) | Reverse (5’-3’) |
| --- | --- | --- |
| HMMR | GACCAGGACUAAUGAACUATT | UAGUUCAUUAGUCCUGGUCTT |
| KIF20A-1# | GCAUCCUUCUUCAACCUAATT | UUAGGUUGAAGAAGGAUGCTT |
| KIF20A-2# | GGAUCUCAUUCUUUGAGAUTT | AUCUCAAAGAAUGAGAUCCTT |
| GPR87 | GCAUCUUGCUGAAUGGUUUTT | AAACCAUUCAGCAAGAUGCTT |
| PGM2L1 | GCUCCGCUGGGAUAAGAAUTT | AUUCUUAUCCCAGCGGAGCTT |
| ANKZF1 | CCAGGGUCCUAUGGAUAUUTT | AAUAUCCAUAGGACCCUGGTT |

| Gene | Forward (5’-3’) | Reverse (5’-3’) |
| --- | --- | --- |
| HMMR | GACCAGGACUAAUGAACUATT | UAGUUCAUUAGUCCUGGUCTT |
| KIF20A-1# | GCAUCCUUCUUCAACCUAATT | UUAGGUUGAAGAAGGAUGCTT |
| KIF20A-2# | GGAUCUCAUUCUUUGAGAUTT | AUCUCAAAGAAUGAGAUCCTT |
| GPR87 | GCAUCUUGCUGAAUGGUUUTT | AAACCAUUCAGCAAGAUGCTT |
| PGM2L1 | GCUCCGCUGGGAUAAGAAUTT | AUUCUUAUCCCAGCGGAGCTT |
| ANKZF1 | CCAGGGUCCUAUGGAUAUUTT | AAUAUCCAUAGGACCCUGGTT |
