# Supplementary material for: A Novel Risk Factor Model Based on Glycolysis-Associated Genes for Predicting the Prognosis of Patients With Prostate Cancer
Source: Front Oncol. 2021 Sep 14;11:605810. doi: 10.3389/fonc.2021.605810 (PMC8476926; doi:10.3389/fonc.2021.605810)
Supplement: Supplementary file 9 [file Table_2.docx]

Table S2. Summary about the gene sets derived from GESA

| Gene sets | Source platform | Contributor | Counts |
| --- | --- | --- | --- |
| BIOCARTA_GLYCOLYSIS_PATHWAY | SEQ_ACCESSION | BioCarta | 3 |
| HALLMARK_GLYCOLYSIS | HUMAN_GENE_SYMBOL | Arthur Liberzon | 200 |
| REACTOME_GLYCOLYSIS | EnsemblGeneIds | Reactome | 72 |
| GLYCOLYSIS_GLUCONEOGENESIS | EntrezGeneIds | KEGG | 62 |
| GLYCOLYTIC_PROCESS | EntrezGeneIds | Gene Ontology | 62 |
